# A Mendelian randomization study of IL6 signaling in cardiovascular diseases, immune-related disorders and longevity

**DOI:** 10.1038/s41525-019-0097-4

**Published:** 2019-09-20

**Authors:** Mickael Rosa, Arnaud Chignon, Zhonglin Li, Marie-Chloé Boulanger, Benoit J. Arsenault, Yohan Bossé, Sébastien Thériault, Patrick Mathieu

**Affiliations:** 1 Laboratory of Cardiovascular Pathobiology, Quebec Heart and Lung Institute/Research Center, Department of Surgery, Laval University, Quebec, Canada; 2 Department of Medicine, Laval University, Quebec, Canada; 3 Department of Molecular Medicine, Laval University, Quebec, Canada; 4 Department of Molecular Biology, Medical Biochemistry and Pathology, Laval University, Quebec, Canada

**Keywords:** Cardiovascular diseases, Genetics research

## Abstract

Growing evidence suggests that inflammation is a significant contributor to different cardiovascular diseases (CVDs). Mendelian randomization (MR) was performed to assess the causal inference between plasma soluble IL6 receptor (sIL6R), a negative regulator of IL6 signaling, and different cardiovascular and immune-related disorders. *Cis*-MR with multiple instrumental variables showed an inverse association of sIL6R with rheumatoid arthritis, atrial fibrillation, stroke, coronary artery disease, and abdominal aortic aneurysm. However, genetically-determined sIL6R level was positively associated with atopic dermatitis and asthma. Also, sIL6R level was associated with longevity, as evaluated by parental age at death, a heritable trait. Gene-based association analysis with S-PrediXcan by using tissues from GTExV7 showed that *IL6R* tissue expression-disease pair associations were consistent with the directional effect of IL6 signaling identified in MR. Genetically-determined reduced IL6 signaling lowers the risk of multiple CVDs and is associated with increased longevity, but at the expense of higher atopic risk.

## Introduction

Immune-regulated processes are involved in the development of several cardiovascular disorders (CVDs), including coronary artery disease (CAD), atrial fibrillation (AF) and stroke. A recent randomized controlled trial (RCT), the Canakinumab Anti-Inflammatory Thrombosis Outcomes Study (CANTOS), showed in high-risk CAD patients that canakinumab, an anti-interleukin 1 beta (IL1B) therapy, reduced major adverse cardiovascular events (MACE).^1^ Post hoc analyses of the CANTOS trial showed that individuals with a reduction of plasma interleukin 6 (IL6) level under anti-IL1B therapy experienced greater reduction of MACE.^2^ These findings raised the hypothesis that canakinumab-mediated modulation of IL6 pathway provides risk-reduction in CAD patients. More recently, a RCT testing low-dose methotrexate did not achieve reduction of primary event rates. In this trial, methotrexate, an immune modulating drug, did not reduce significantly the plasma level of IL1B, IL6, and C-reactive protein (CRP), a sensitive biomarker of IL6 signaling.^3^

IL6 is a pleiotropic cytokine and its circulating level has been identified as a prognostic marker for different CVDs such as AF and CAD.^4,5^ In the last several years, different biologically-derived drug compounds have been developed to inhibit IL6 signaling. For instance, tocilizumab, which targets IL6 signaling, has been approved for the treatment of rheumatoid arthritis (RA).^6^ In the blood plasma, a soluble fraction of the IL6 receptor (sIL6R) forms with soluble gp130 (sgp130/sIL6ST) and IL6 an inhibitory complex.^7^ As such, plasma sIL6R, which is acting as a decoy receptor, negatively regulates IL6 signaling. At the tissue level, however, the expression of IL6R, which is recruited to a cell membrane protein complex, promotes IL6 signaling.^8^ The level of IL6R is largely under the control of genetic factors.^9^ Genome-wide association studies (GWAS) for CAD have consistently detected a genome-wide significant signal in the *IL6R* locus.^10,11^ In AF, studies with a targeted approach have identified gene variants with nominally significant *p* values in the *IL6R* locus.^12,13^ The use of genetic variants as instrumental variables (IVs) in Mendelian randomization (MR) eliminates reverse causation bias and allows causal inference. Three main assumptions for the IVs must be satisfied: (1) the genetic variants must be associated with the risk factor, (2) the genetic variants must not be associated with confounders of the relation between the risk factor and the outcome, and (3) the genetic variants must be associated with the outcome only through the risk factor.^14^ Previous work using a single genetic variant as IV, rs7529229, showed that circulating level of soluble interleukin-6 receptor (sIL6R) was associated with CAD.^15^ Gene variant rs7529229 is in linkage disequilibrium (LD) (*r*^2^ = 0.97) with rs2228145, a non-synonymous variant that increases the cleavage of membrane IL6R and, therefore, is positively associated with plasma sIL6R level. Recently, a phenome-wide analysis based on rs2228145 identified an association signal with abdominal aortic aneurysm (AAA).^16^ MR using a single variant as IV has inherent important limitations, including among others the risk that the variant, or another one in LD, affects the outcome directly or through another route (pleiotropy), thus violating MR assumptions. The use of multiple *cis*-acting gene variants as independent and strong IVs increases power, precision and minimizes the risk of pleiotropy.^14^

In the present work, we aimed to determine causal associations between sIL6R and complex trait disorders with an inflammatory component. We leveraged recent GWAS summary-level data for circulating sIL6R and we identified 34 independent genetic variants as IVs that were used in MR. Mapping of large GWAS summary-level data including more than one million individuals showed that genome-wide significant variants in *IL6R* are associated with AF. Multi-trait analyses showed a causal role for IL6 signaling in different disorders including causal associations with AF and stroke, and also identified causal associations with atopic disorders. In addition, we highlighted that genetically-determined blood plasma sIL6R level was causally associated with parental age at death, a transmissible trait related to longevity.

## Results

### Soluble IL6R lowers systemic inflammation as measured by CRP

**Fig. 1:**
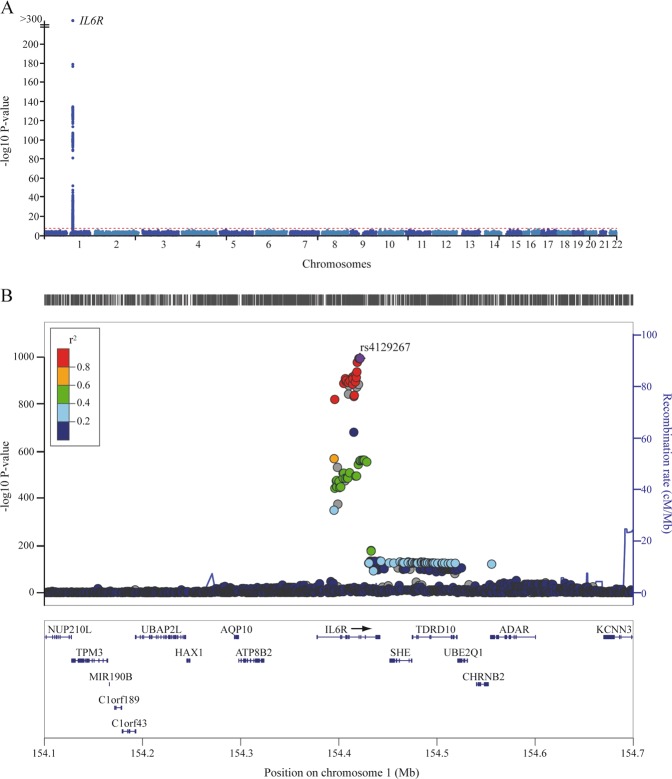
GWAS for plasma sIL6R. **a** Manhattan plot showing the GWAS for plasma sIL6R levels. The *y*-axis represents *P*_GWAS_ in −log10 scale. The horizontal red dot line indicates the *P*_GWAS_ significance threshold of 5 × 10^−8^. **b** Regional plot of gene variants and their associations with sIL6R level. Genes located 200 kb up- and downstream of *IL6R* are displayed below the plot

**Fig. 2:**
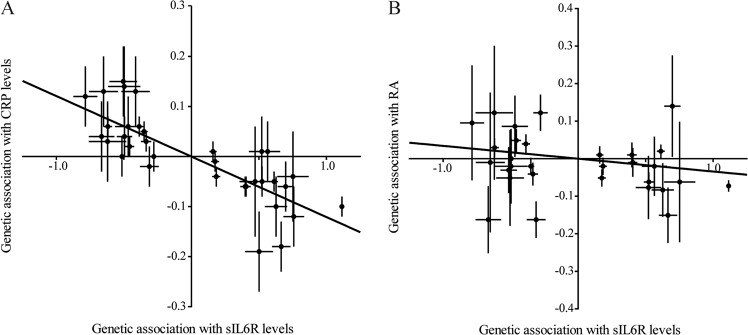
sIL6R causal association with inflammation. Mendelian randomization analysis of the association between circulating sIL6R and: **a** CRP levels, **b** rheumatoid arthritis (RA) risk. Each dot represents an independent gene variant located within 250 kb of *IL6R* and selected for its association with sIL6R levels (*r*^2^ < 0.1, *F*-statistic > 15)

GWAS summary-level data from 17 different disorders-traits were assembled to assess the putative role of IL6 signaling. We leveraged a recent and extensive GWAS for 2994 blood plasma proteins performed in 3301 individuals of European ancestry.^17^ Measurements were performed with SOMAmers, a validated high-throughput and aptamer-based method. Figure 1a, b shows the Manhattan and locus plots for circulating sIL6R and identifies the strongest and genome-wide significant signal at 1q21.3, which includes *IL6R*. The lead variant in this region is rs4129267 (*P*_GWAS _ = 7.41 x 10^−1101^), which is in perfect LD (*r*^2^ = 1) with the non-synonymous variant rs2228145. In this region, we identified 34 independent *cis*-acting gene variants (*r*^2^ < 0.1, *F-*statistic > 15) as IVs for plasma sIL6R level, which were located within 250 kb from *IL6R* (Supplementary Table 1). Two-sample MR by using *cis*-acting variants as IVs for sIL6R was first assessed on the plasma levels of high-sensitivity C-reactive protein (hs-CRP) and serum amyloid A (SAA), two biomarkers downstream of IL6 signaling measured in cohorts of 9961 and 3301 subjects respectively. MR analysis showed inverse causal inference for sIL6R with plasma hs-CRP level (Beta per 1 SD: −0.10; 95% CI, −0.12 to −0.08; *P*_causal_ = 7.33 × 10^−24^) (Fig. 2a). However, sIL6R was not causally associated with SAA (Beta: −0.02; 95% CI: −0.06–0.01; *P*_causal_ = 0.14). As sIL6R seems causally linked with lower level of hs-CRP, a sensitive biomarker of inflammation, we reasoned that the level of sIL6R may mimic anti-IL6 therapy. Tocilizumab, which lowers IL6 signaling, is approved for the treatment of RA. We thus assessed the relationships between circulating sIL6R and RA in a cohort of 67,860 individuals. MR analysis showed an inverse and causal association between sIL6R and RA (Odd ratio [OR]: 0.95; 95% CI: 0.93–0.98; *P*_causal_ = 9.55 × 10^−4^) **(**Fig. 2b**)**. These findings indicate that genetically-determined elevated plasma sIL6R level reduces systemic inflammation and decreases the risk of RA, a disease genetically-correlated to different CVDs (stroke, *r*_g_ = 0.19, *P* = 0.003; AF, *r*_g_ = 0.08, *P* = 0.01) and with a strong immune component.

### Soluble IL6R and cardiovascular disorders

**Fig. 3:**
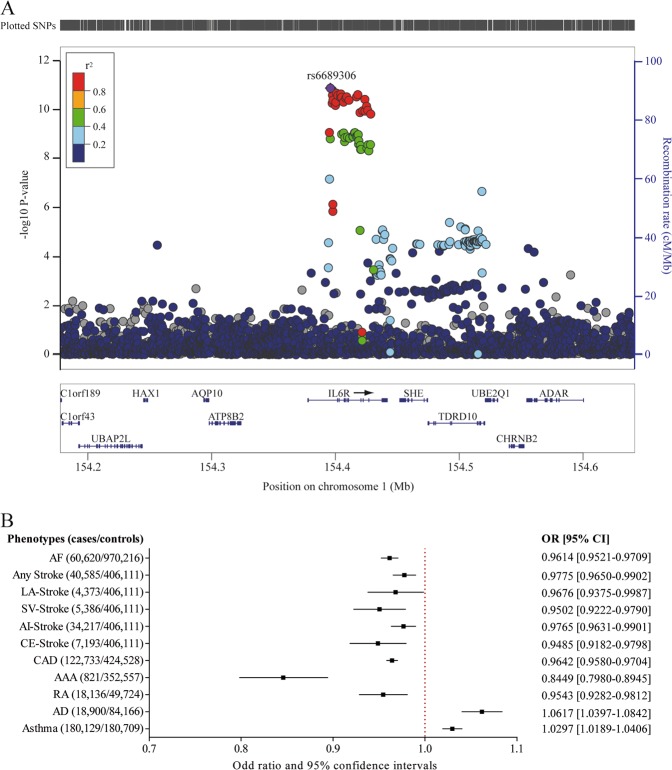
Gene mapping for atrial fibrillation and causal associations of sIL6 with eleven disorders. **a** Regional plot of the genetic variants at 1q21.3 and their association with AF. **b** Forest plot of sIL6R MR showing the OR and 95% confidence interval (95% CI) for each disorder. AF atrial fibrillation, CAD coronary artery disease, LA-stroke large artery stroke, SV-stroke small vessel stroke, AI-stroke any ischemic stroke, CE-stroke cardioembolic stroke, AAA abdominal aortic aneurysm, RA rheumatoid arthritis, AD atopic dermatitis

Growing evidence suggests that inflammation may play a role in the pathogenesis of AF, but whether immune-related processes are causal in the genesis of arrhythmia is presently unknown. By using summary statistics of a recent GWAS including 60,620 cases and 970,216 controls, we assessed the putative role of IL6 signaling on the risk of AF. Positional mapping of genetic associations for AF identified a strong signal in *IL6R* (Fig. 3a), which was not mapped in previous GWAS analysis; the reason is likely that previous mapping identified at this locus *KCNN3*, a well-known AF gene with a very strong signal (Supplementary Fig. 1). The index variant in AF is rs6689306 (*P*_GWAS_ = 1.36 × 10^−11^), which is intronic to *IL6R* and in moderate LD (*r*^2^ = 0.35) with rs4129267, the top lead variant associated with plasma sIL6R level. In MR, we found a strong, inverse and causal inference between sIL6R and AF risk (OR: 0.96; 95% CI: 0.95–0.97; *P*_causal_ = 3.64 × 10^−15^) (Fig. 3b). Mapping of GWAS data for AF showed a significant enrichment for stroke (*P*_FDR_ = 2.22 × 10^−4^) in the GWAS catalog. As AF is a risk factor for stroke, we assessed the relationship with sIL6R in MR analysis. Data were restricted to individuals of European ancestry from a recent multi-ancestry GWAS for stroke (any stroke) and including four different stroke subtypes: large artery (LA-stroke), small vessel (SV-stroke), any ischemic (AI-stroke) and cardio-embolic (CE-stroke) strokes. MR analysis showed an inverse and causal inference between sIL6R and stroke (any stroke) (OR: 0.98; 95% CI: 0.97–0.99; *P*_causal_ = 5.64 × 10^−4^) (Fig. 3b). Moreover, sIL6R was also inversely and causally associated with every stroke subtypes (LA-stroke, OR: 0.968; 95% CI: 0.938–0.999; *P*_causal_ = 0.041; SV-stroke, OR: 0.95; 95% CI: 0.92–0.98 *P*_causal_ = 8.13 × 10^−4^; AI-stroke, OR: 0.98; 95% CI: 0.96–0.99; *P*_causal_ = 7.38 × 10^−4^; and CE-stroke, OR: 0.95; 95% CI: 0.92–0.98; *P*_causal_ = 1.42 × 10^−3^) (Fig. 3b). We next performed mediation analyses in multivariable MR to evaluate the implication of AF on the risk of stroke. As expected, the relationship between sIL6R and CE-stroke was not significant (OR: 0.97; 95% CI: 0.92–1.03; *P*_causal_ = 0.30) after controlling for the association with AF. However, after controlling for the association with AF, sIL6R remained associated with any stroke (OR: 0.97; 95% CI: 0.95–0.99; *P*_causal_ = 0.01). Next, as MR analysis based on a single genetic variant suggested a role for IL6 signaling in CAD, we assessed the causal association with multiple IVs in a recent meta-analysis totaling 547,261 individuals. This analysis showed a highly significant, inverse and strong causal inference between sIL6R and CAD (OR: 0.964; 95% CI: 0.958–0.970; *P*_causal_ = 6.98 × 10^−29^) (Fig. 3b). To rule out a possible effect of IL6 signaling on blood lipid level, a causal risk factor for CAD, genetic association data on plasma lipid levels were assembled for MR analysis with individual data from UKB: high-density lipoprotein (HDL, *n* = 255,929), low-density lipoprotein (LDL, *n* = 279,367), total cholesterol (*n* = 279,913) and triglycerides (*n* = 279,686). This analysis revealed association between sIL6R and HDL (Beta: 0.005; 95% CI: 0.001–0.010; *P*_causal_ = 0.020) and between sIL6R and total cholesterol (Beta: 0.005; 95% CI: 0.001–0.008, *P*_causal_ = 4.74 × 10^−3^). There was no association between sIL6R and LDL nor with triglycerides levels (LDL: Beta: 0.002, 95% CI −0.001–0.005, *P*_causal_ = 0.197; triglycerides: Beta: −0.002; 95% CI: −0.005–0.001; *P*_causal_ = 0.214). In mediation analysis by using multivariable MR the association between sIL6R and CAD remained significant after controlling for HDL or total cholesterol (OR: 0.963; 95% CI: 0.956–0.970; *P*_causal_ *<* 0.0001 and OR: 0.962; 95% CI: 0.955–0.969; *P*_causal_ *<* 0.0001 respectively). GWAS meta-analysis for CAD from van der Harst et al.^10^ includes patients from UKB. When restricting multivariable MR analysis corrected for lipids to CARDIOGRAMplusC4D data (CAD GWAS), we obtained similar results with sIL6R remaining associated with CAD (Supplementary Table 2). Finally, we tested the association of IL6 signaling in AAA, an atherothrombotic vascular disorder. By using individual data from UKB (*n* = 353,378, including 821 cases), we found an inverse causal inference between sIL6R and AAA (OR: 0.85; 95% CI: 0.80–0.90; *P*_causal_ = 7.08 × 10^−9^) (Fig. 3b).

### Phenome-wide association study ((PheWAS) for IL6 signaling

**Fig. 4:**
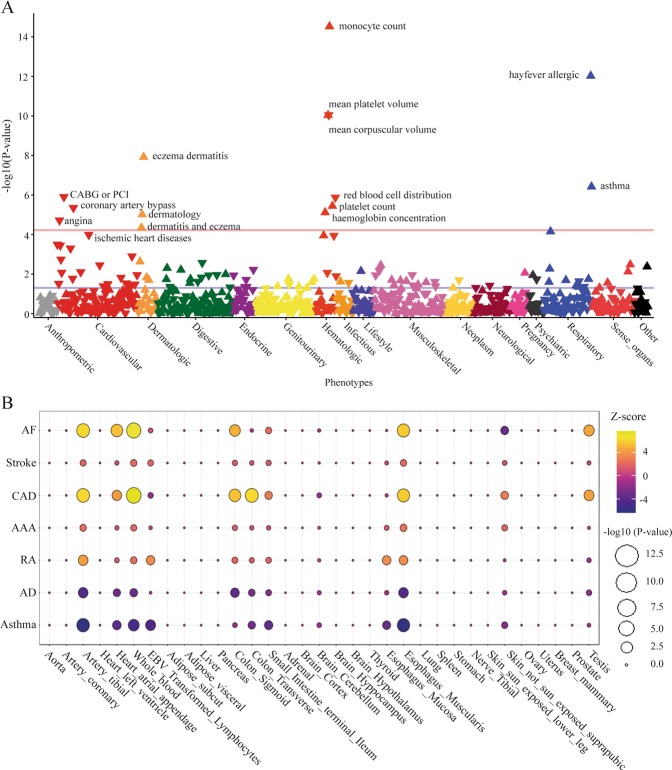
PheWAS and S-Predixcan analysis. **a** PheWAS for rs4129267. **b** Balloon plot illustrating *IL6R* tissue expression-disease association pairs; size of circle is proportional to –log10 P-value, whereas the color indicates the Z-score

We next performed a PheWAS in UKB to examine possible relationships between sIL6R and other diseases-traits. The PheWAS reveals that the top sIL6R variant, rs4129267 (*P*_GWAS _ = 7.41 × 10^−1101^), was associated with monocyte count *(P*_PheWAS _ = 2.97 × 10^−15^), mean platelet volume (MPV) (*P*_PheWAS_ = 9.03 × 10^−11^), and atopic disorders such as atopic dermatitis/eczema (AD) (*P*_PheWAS *=* _1.19 × 10^−8^) and asthma (*P*_PheWAS _ = 3.79 × 10^−7^) (Fig. 4a). Thus, we assessed causal relationships for plasma sIL6R in AD and asthma in two large cohorts of 103,066 and 360,838 individuals respectively. MR analysis showed positive and causal inference between sIL6R and AD (OR: 1.06, 95% CI: 1.04–1.08; *P*_causal _ = 2.01 × 10^−8^) as well as with asthma (OR: 1.03; 95% CI: 1.02–1.04; *P*_causal _ = 5.62 × 10^−8^) (Fig. 3b).

### Impact of sIL6R on longevity

Considering the importance of IL6 signaling as a causal factor on multi-traits/disorders we hypothesized that plasma level of sIL6R could affect longevity. Assuming that longevity is partially heritable, we assessed the impact of genetically-determined blood plasma sIL6R on parental age at death, an indirect measure of longevity.^18^ Summary statistics from UKB for fathers (*n* = 248,726) and mothers (*n* = 199,690) age at death were used for this analysis. In MR, genetically-determined blood plasma sIL6R was positively associated with fathers age at death (Beta: 0.007; 95% CI: 0.004–0.010, *P*_causal _ = 3.83 × 10^−5^) and mothers age at death (Beta: 0.008; 95% CI: 0.004–0.012; *P*_causal _ = 2.11 × 10^−5^) (Table 1).

**Table 1:** Mendelian Randomization analyses for cardiovascular and immune disorders, blood parameters and longevity

				IVW			
Phenotypes	Cases	Controls	IVs	OR	95% Confidence interval		*P*_causal_
AF	60,620	970,216	34	0.961	0.952	0.971	3.64 × 10^−15^
Any stroke	40,585	406,111	33	0.978	0.965	0.990	5.64 × 10^−4^
LA-stroke	4373	406,111	33	0.968	0.938	0.999	0.041
SV-stroke	5386	406,111	33	0.950	0.922	0.979	8.13 × 10^−4^
AI-stroke	34,217	406,111	33	0.977	0.963	0.990	7.38 × 10^−4^
CE-stroke	7193	406,111	34	0.949	0.918	0.980	1.42 × 10^−3^
CAD	122,733	424,528	34	0.964	0.958	0.970	6.98 × 10^−29^
AAA	821	352,557	34	0.845	0.798	0.895	7.08 × 10^−9^
RA	18,136	49,724	30	0.954	0.928	0.981	9.55 × 10^−4^
AD	18,900	84,166	34	1.062	1.040	1.084	2.01 × 10^−8^
Asthma	180,129	180,709	28	1.030	1.019	1.041	5.62 × 10^−8^

Blood Parameters	Participants		IVs	Beta	95% Confidence interval		*P*_causal_
HDL	255,929		34	0.005	0.001	0.010	0.020
LDL	279,367		34	0.002	−0.001	0.005	0.197
Total cholesterol	279,913		34	0.005	0.001	0.008	4.74 × 10^−3^
Triglycerides	279,686		34	−0.002	−0.005	0.001	0.214
CRP	9961		34	−0.102	−0.122	−0.082	7.33 × 10^−24^
SAA	3301		34	−0.024	−0.055	0.008	0.144

Longevity	Participants		IVs	Beta	95% Confidence interval		*P*_causal_
Fathers age at death	248,726		34	0.007	0.004	0.010	3.83 × 10^−5^
Mothers age at death	199,690		34	0.008	0.004	0.012	2.11 × 10^−5^

*AF* atrial fibrillation, *LA* large artery, *SV* small vessel, *AI* any ischemic, *CE* cardio embolic, *CAD* coronary artery disease, *AAA* abdominal aortic aneurysm, RA rheumatoid arthritis, *AD* atopic dermatitis, *IVW* inverse variance weighted, MR Mendelian randomization

### Tissue expression of *IL6R* and disease-associated risk

*IL6R* expression in tissues may also drive the risk in different disorders. It is worth highlighting that, in tissue, a higher expression of IL6R is related to increased signaling.^8^ By using 48 tissues-cells in GTExV7 and S-Predixcan, we assessed tissue-based expression level and associations with disorders in whom IL6 signaling was causally associated. Figure 4b shows the associations between the expression of *IL6R* in tissues and different disorders. Several tissues including relevant tissues/organs for CVDs such as tibial artery, atrial appendage and whole blood were significantly associated with the risk. The direction of the effect (Z-score) is consistent with MR analyses. In this regard, for CVDs, MR data suggested a positive and causal role for increased IL6 signaling. This is reflected in the expression of *IL6R* in different tissues, which showed positive associations with CVD risk. In contrast, in atopic disorders, the expression of *IL6R* in different tissues was negatively associated with the risk, which is consistent with MR analyses showing a protective role for IL6 signaling in these pathologies.

### Sensitivity analyses

Different sensitivity analyses were performed for sIL6R and causal associations with disorders. Sensitivity analyses by using Egger regression showed that causal associations with sIL6R remained significant and without horizontal pleiotropy for AF (OR: 0.95; 95% CI: 0.93–0.97; *P*_Egger_ = 4.82 × 10^−7^, *P*_intercept_ = 0.22), CE-stroke (OR: 0.93; 95% CI: 0.87–0.99; *P*_Egger_ = 0.03, *P*_intercept_ = 0.53), CAD (OR: 0.96; 95% CI: 0.95–0.97; *P*_Egger_ = 9.23 × 10^−11^, *P*_intercept_ = 0.31), AAA (OR: 0.82; 95% CI: 0.73–0.92; *P*_Egger_ = 8.86 × 10^−4^, *P*_intercept_ = 0.58), RA (OR: 0.94; 95% CI: 0.89–0.99; *P*_Egger_ = 0.02, *P*_intercept_ = 0.50), AD (OR: 1.08; 95% CI: 1.04–1.13; *P*_Egger_ = 2.29 × 10^−4^, *P*_intercept_ = 0.34), asthma (OR: 1.04; 95% CI: 1.01–1.06; *P*_Egger_ = 1.22 × 10^−3^, *P*_intercept_ = 0.54) and fathers age at death (Beta: 0.007; 95% CI, 0.001–0.014; *P*_Egger_ = 0.032, *P*_intercept_ = 0.87) (Supplementary Table 3). To assess robustness of the results, weighted median method was also performed.^19^ This analysis showed that the associations between sIL6R and AF (OR: 0.96; 95% CI: 0.95-0.98; *P*_causal _ = 1.05 × 10^−10^), any stroke (OR: 0.979; 95% CI: 0.964–0.995; *P*_causal _ = 8.91 × 10^−3^), SV-stroke (OR: 0.96; 95% CI: 0.92–0.99; *P*_causal _ = 0.02), AI-stroke (OR: 0.980; 95% CI: 0.963–0.997; *P*_causal _ = 0.018), CE-stroke (OR: 0.96; 95% CI: 0.93–0.99; *P*_causal _ = 7.10 × 10^−3^), CAD (OR: 0.965; 95% CI: 0.957–0.973; *P*_causal _ = 1.54 × 10^−16^), AAA (OR: 0.85; 95% CI: 0.78–0.92; *P*_causal _ = 7.29 × 10^−5^), RA (OR: 0.94, 95% CI: 0.92–0.97; *P*_causal _ = 5.03 × 10^−6^), AD (OR: 1.07, 95% CI: 1.04–1.10; *P*_causal _ = 2.01 × 10^−6^) asthma (OR: 1.03; 95% CI: 1.02–1.04; *P*_causal _ = 3.65 × 10^−11^) and father age at death (Beta: 0.006; 95% CI: 0.001–0.011; *P*_causal _ = 0.010) remained significant (Supplementary Table 3). Additional sensitivity analyses were performed by removing the lead variant associated with sIL6R. After removing the top variant rs4129267, we found that MR analysis remained significant for AF (OR: 0.96; 95% CI: 0.95–0.97; *P*_causal_ = 4.37 × 10^−10^), any stroke (OR: 0.98; 95% CI: 0.96–0.99; *P*_causal_ = 6.83 × 10^−3^), LA-stroke (OR: 0.955; 95% CI: 0.916–0.997 *P*_causal_ = 0.034), SV-stroke (OR: 0.94; 95% CI: 0.91–0.98; *P*_causal_ = 5.14 × 10^−3^), AI-stroke (OR: 0.97; 95% CI: 0.96–0.99; *P*_causal_ = 5.88 × 10^−3^), CE-stroke (OR: 0.94; 95% CI: 0.90–0.99; *P*_causal_ = 7.98 × 10^−3^), CAD (OR: 0.963; 95% CI: 0.955–0.972; *P*_causal_ = 2.49 × 10^−16^), AAA (OR: 0.84; 95% CI: 0.78–0.91; *P*_causal _ = 5.30 × 10^−6^), AD (OR: 1.06; 1.02–1.09; *P*_causal_ = 3.49 × 10^−4^), asthma (OR: 1.03; 95% CI, 1.01–1.04; *P*_causal_ = 2.93 × 10^−4^), fathers age at death (Beta: 0.008; 95% CI: 0.003–0.012; *P*_causal_ = 4.69 × 10^−4^) and mothers age at death (Beta: 0.011; 95% CI: 0.006–0.016; *P*_causal_ = 5.19 × 10^−6^) (Suppl. Table 3). After removing the top sIL6R variant, the causal association between sIL6R and RA was not significant (*P*_causal_ = 0.27) (Supplementary Table 3). A recent study suggested that MR can be reinforced by performing colocalization analysis.^20^ Colocalization analyses showed that sIL6R signal was shared with AAA (PP4 = 88%), AD (PP4 = 98%) and asthma (PP4 = 99.2%) (Supplementary Table 4).

## Discussion

**Fig. 5:**
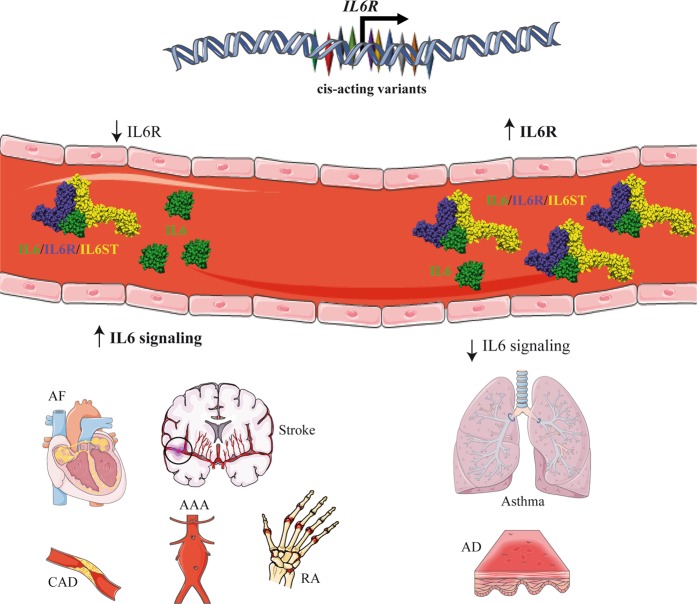
Schematic representation of IL6 signaling in different disorders. Gene variants impact the level of soluble interleukin-6 (sIL6R), which inhibits free IL6 in the bloodstream by forming a complex with soluble gp130 (sgp130/sIL6ST). On the one hand, genetically-determined lower level of circulating sIL6R increases the bioavailability of IL6, which, in turn, promotes the development of cardiovascular disorders and rheumatoid arthritis. On the other hand, genetically-determined higher circulating sIL6R lower IL6 signaling and promotes atopic disorders. This figure was created with images adapted from Servier Medical Art by Servier (https://smart.servier.com/). Original images are licensed under a Creative Commons Attribution 3.0 Unported License. AF atrial fibrillation, CAD coronary artery disease, AAA abdominal aortic aneurysm, RA rheumatoid arthritis, AD atopic dermatitis

Consistent with biological data, the present findings corroborate a potential causal role for sIL6R in lowering hs-CRP plasma level.^15^ By using a robust two-sample MR approach, which included multiple strong and independent IVs as well as different sensitivity analyses, we found causal associations for IL6 signaling in AF and stroke. Also, we confirmed previous causal association for IL6 signaling in CAD and we extended these data to AAA, an atherothrombotic vascular disorder. PheWAS and MR analyses also unraveled a protective role for IL6 signaling in AD and asthma (Fig. 5). Genetically determined blood plasma sIL6R was also positively associated with parental age at death.

Previous work has underlined that in circulation sIL6R forms a complex with sgp130/IL6ST and inhibits IL6.^21^ IL6 has widespread and diverse biological actions. In the liver, IL6 promotes the expression of acute phase proteins such as SAA and CRP.^22^ While we did not find a causal inference between genetically determined sIL6R level and SAA, we found an inverse and significant relationship with hs-CRP level in circulation. These findings are consistent with a decreased signaling of IL6 when plasma sIL6R is elevated. Hence, it supports the fact that in circulation, sIL6R is acting as a decoy receptor to neutralize IL6. Consistently, we underlined that genetically elevated plasma sIL6R levels decreased the risk of RA, an immune-related disorder in which the role of IL6 signaling is well established.^23^

Circulating level of IL6 has been associated with different CVDs and outcomes. For instance, plasma IL6 level has been positively associated with the risk of AF and left atrial dimension.^24^ Candidate gene approaches have identified nominally significant gene variants in *IL6R* that were associated with AF.^12,13^ In the present work, we leveraged summary-level data of a large GWAS with more than a million individuals to map *IL6R* to AF. Positional mapping identified a genome-wide significant variant, rs6689306, located in the intron of *IL6R* and associated with AF. MR with multiple *cis*-acting gene variants as IVs showed a robust causal inference between circulating sIL6R and AF. Genetically-determined higher sIL6R level lowered the risk of AF. IL6 has been shown to induce cardiac fibrosis and remodeling.^25^ Whether IL6 promotes AF through a remodeling process of the left atrium remains, however, to be investigated. In line with the present findings in AF, we found a potential causal role for IL6 signaling in CE-stroke. Mediation analysis showed that the risk of CE-stroke was largely driven by the association between IL6 signaling and the risk of AF. However, it is worth pointing out that our analyses revealed causal inference for IL6 signaling with any stroke. In multivariable MR, the causal inference between sIL6R and any stroke remained significant despite controlling for the association with AF. These data suggest that IL6 signaling is likely associated in a causal manner with the risk of stroke and this beyond the risk of AF. Previous MR based on a single genetic variant as IV suggested a causal association between sIL6R and CAD.^15^ In the present work, by using multiple *cis*-acting and independent variants as IVs, we confirmed in MR previous analyses and we demonstrate a strong and inverse causal inference between sIL6R and CAD risk. Also, we found a strong association between increased IL6 signaling and the risk of AAA. These data are in line with a recent meta-analysis of GWAS for AAA, which identified a genome-wide significant signal at rs4129267, the top variant in *IL6R* associated with the plasma level of sIL6R.^26^ At the tissue level, gene-based expression analysis for the different disorders highlighted consistent associations with IL6R signaling. For CVDs, we observed with S-PrediXcan positive Z-scores in different *IL6R* tissue expression-disease pair associations. In tissues, a higher expression of *IL6R*, which forms a cell membrane complex responsive to circulating IL6, promotes IL6 signaling.^8^ Taken together, these data strongly suggest positive and causal relationships between IL6 signaling and the risk of four major CVDs associated with significant morbidity and mortality: AF, stroke, CAD, and AAA.

Previous studies identified genome-wide significant signals at *IL6R* in atopic disorders.^27^ By using individual data from UKB in a PheWAS, we confirmed previous findings and found significant associations between sIL6R top variant and atopic disorders. In MR, genetically determined plasma sIL6R level was positively and causally associated with AD and asthma, two disorders with significant genetic correlation, which often co-occur in patients. The reason why sIL6R is causally associated with CVDs and atopic disorders, but with opposite directional effect, is not clear. However, it is likely that IL6 signaling, which may have pro- and anti-inflammatory activity, has different interplays with CVDs and atopic disorders. In a mouse model, the administration of IL6 decreased Th2 cytokines and ameliorates asthma.^28^ In patients, tocilizumab, an anti-IL6 signaling therapy, has been associated with some adverse events including skin rashes and eczema.^29^

The present investigation has potential important clinical implications. MR analysis using strong IVs identified robust causal associations between IL6 signaling and CVD risk. Novel associations suggest that modulating the action of IL6 could reduce the risk of AF and stroke. Moreover, we confirmed and extended previous data in showing strong causal inference for IL6 signaling with CAD and AAA risk. Further analyses also suggested that lower IL6 signaling is linked to increased longevity. To this effect, genetically-predicted blood plasma sIL6R, which negatively regulates IL6 signaling, seemed positively and causally associated with parental age at death, a heritable trait.^18^ Hence, targeting this pathway could provide meaningful and substantial CVD risk reduction. Different therapeutic agents targeting IL6 are in development or approved for different indications such as RA. To this effect, tocilizumab, which blocks IL6 signaling, is approved for the treatment of RA. One side effect of tocilizumab is to increase serum cholesterol level.^6^ The present analyses showed that sIL6R was positively associated with plasma HDL and total cholesterol. However, these associations were not significant in Egger MR and after removing the top variant (Supplementary Table 3). Nonetheless, in multivariate MR the association between sIL6R and CAD was independent from lipids. Hence, further work is needed to tease out the role of IL6 signaling on lipid metabolism. It is worth highlighting that other anti-IL6 therapy are under development such as anti-IL6 antibody or humanized sgp130FC and could represent suitable agents to test in eventual RCTs. Recent work also suggests that when combined to MR strong colocalization between plasma protein quantitative trait loci (pQTL) and diseases may help predict future drug approval.^20^ We found strong colocalization signal for sIL6R with AAA and atopic disorders. Hence, present findings also warrant that caution should be used in the design of eventual clinical trials that would examine IL6-based therapy for CVDs, especially with regard to atopic patients.

Present MR analyses suggest causal associations between IL6 signaling and CVDs. Different sensitivity analyses indicate that causal inference associations were robust. In Egger analyses, there was no horizontal pleiotropy for the different associations. Analyses without the top sIL6R variant showed persistent and consistent causal estimates for CVDs. Hence, these data strongly suggest that modulating IL6 signaling may decrease substantially cardiovascular risk. However, it is worth pointing out that despite strong causal inference, it is only through RCT that causality can be firmly established.

By leveraging recent GWAS data we highlighted that targeting IL6 and its signaling pathway could decrease AF, stroke, CAD, and AAA. Causal inferences were robust to different sensitivity analyses and they strongly militate for the implication of IL6 in different CVDs. We found opposite directional effect for IL6 signaling between CVDs and atopic disorders. Hence, it is possible that anti-IL6 therapy, which has, so far, shown an adequate security profile, may increase atopic risk in some susceptible individuals. Further work, including RCTs, should evaluate anti-IL6 based therapy on cardiovascular outcomes.

## Methods

### Publicly available data

We performed analyses based on 17 publicly available GWAS summary statistics. As all analyses were based on publicly available summary statistics, no ethical approval was required.

### Summary statistics for sIL6R and Serum Amyloid A

GWAS data with individuals of European ancestry (*n* = 3301), which evaluated the plasma proteome (*n* = 2994 proteins) with SOMAmer technology,^17^ was leveraged for MR analyses. Results were adjusted for age, sex, duration between blood draw and processing and for the first three principal components of ancestry from multi-dimensional scaling. The two subcohorts of the study were combined in fixed-effects inverse variance meta-analysis. After adjustment, 10,572,788 variants were suitable for analysis.^17^

### Summary statistics for high-sensitivity CRP

Summary-level data including 9961 individuals of European ancestry were analyzed for high-sensitivity CRP plasma levels.^30^ Data were adjusted for age and sex. Inverse variance weighted and fixed-effects meta-analysis was performed. Variants with MAF < 0.01 were excluded resulting in 9,391,608 variants available for analyses.^30^

### Summary statistics for lipids

Data for HDL, LDL, total cholesterol and triglycerides levels were obtained from individual data from UK Biobank (255,929 subjects for HDL; 279,367 for LDL; 279,913 for total cholesterol and 279,686 for triglycerides). Adjustment for age, sex and the 10 first principal components was performed.

### Summary statistics for AF

The data analyzed consisted in the GWAS summary statistics of 60,620 cases of AF and 970,216 controls of European ancestry.^31^ Data were adjusted for age, sex and the 4 first principal components. Meta-analysis was performed by fixed-effects and inverse variance method and variants with minor allele frequency (MAF) < 2.5 × 10^−5^ were excluded (34,740,186 for analyses).^31^ Cases were defined as patients with at least 1 specific code for AF based on ICD 10 codes (“I48” or “427.3”).

### Summary statistics for stroke

The consortium MEGASTROKE genotyped 40,585 cases of strokes divided into any stroke (AS), cardio-embolic stroke (CES), large artery stroke (LAS), acute ischemic stroke (AIS) or small vessels stroke (SVS) and 406,111 controls of European ancestry.^32^ Data were adjusted for age and sex and a fixed-effects meta-analysis was performed. Variants with a MAF < 0.01 were excluded resulting in 8,255,862; 8,306,092; 8,451,007; 8,340,186; 8,765,830 variants available for analyses, respectively for AS, CES, LAS, AIS or SVS. Stroke was defined as patient developing signs of focal or global disturbance of cerebral function lasting for more than 24 h or leading to death. Ischemic stroke definition was based on clinical or imaging criteria. Subdivision in cardioembolic ischemic stroke (CES), LAS or small vessel ischemic stroke (SVS) was performed according to the TOAST criteria.^33^

### Summary statistics for CAD

A meta-analysis including 122,733 cases of CAD and 424,528 controls of European ancestry was analyzed.^10^ Adjustment for age, sex and the first 30 principal components was performed. The different cohorts were meta-analyzed by fixed-effects and inverse variance method and 7,947,838 variants remained after corrections.^10^ CAD was defined using the ICD 10 codes: I21-I25 for ischemic heart diseases and the following OPCS-4 codes: K40-K46, K49, K50, and K75 including transluminal balloon angioplasty, and other therapeutic transluminal operations on coronary artery and percutaneous transluminal balloon angioplasty and insertion of stent into coronary artery. Self reports from patients were also included in the definition with following items: heart attack or myocardial infarction, coronary angioplasty with or without stent and coronary artery bypass grafting. In CARDIoGRAMplusC4D dataset, cases of CAD were defined as patients with myocardial infarction validated by cardiologists and/or a stenosis of at least 50% in one coronary artery.^34^

### Summary statistics for aortic abdominal aneurysm

Data from UK Biobank were used to generate summary statistics for aortic abdominal aneurysm (821 cases and 352,557 controls). Adjustment for age, sex and the 10 first principal components was performed. Variants with a MAF < 0.01 were excluded (13,791,469 variants remaining for analyses).

### Summary statistics for RA

GWAS summary statistics including 18,136 cases of RA and 49,724 controls of European ancestry were analyzed.^35^ Data were adjusted on the first five principal components and were meta-analyzed by inverse variance method assuming a fixed-effects model. Variants with a MAF < 0.01 were excluded (8,747,964 variants available for analyses).^35^ All RA cases were diagnosed by a professional rheumatologist or fulfilled the criteria of the American College of Rheumatology for RA diagnosis.^35^

### Summary statistics for atopic dermatitis

GWAS for atopic dermatitis and controls (18,900 cases and 84,166 controls) was analyzed.^36^ Data were adjusted for ancestry, method of case definition and age of onset. Fixed-effects meta-analysis was performed. Variants with MAF < 0.01 were excluded, leaving 15,539,996 remaining variants for analyses.^36^

### Summary statistics for asthma

The data analyzed consisted in the GWAS summary statistics of 180,129 cases of asthma and 180,709 controls of European ancestry.^37^ Data were adjusted for age and sex. Results of the subcohorts were combined in inverse variance weighted, fixed-effects meta-analysis. A threshold of 0.01 for the MAF was used to exclude variants with lower frequencies (8,307,659 remaining variants for analyses).^37^ Cases were defined as patients reporting for asthma from touchscreen questionnaire or were self-reported during verbal interview.

### Analyses of fathers and mothers age at death as a proxy for longevity assessment

Summary-level data from UK Biobank provided by the Neale laboratory encompassed 248,726 and 199,690 individuals of European ancestry for fathers and mothers age at death respectively. Adjustment for sex was performed. Variants with a MAF < 0.01 were excluded resulting in 13,791,469 variants available for analyses.

### Mapping of GWAS summary statistics

GWAS for plasma sIL6R and AF were mapped to genes by using Functional Mapping and Annotation of GWAS (FUMA).^38^ Gene variants in associated genomic loci with a r^2 ^ ≥ 0.6 and suggestive associations (*P*_GWAS _ < 1 × 10^−5^) with independent significant single nucleotide polymorphisms (SNPs) and MAF ≥ 0.01 were annotated. We performed positional mapping, which mapped gene variants to genes based on physical distance from protein-coding genes (within 10 kb) in the GRCh37/hg19 reference assembly. Gene variants mapped by FUMA were matched with reported SNPs in the GWAS catalog and data reported as enrichment *p*-value (corrected *P*_FDR_) for a given trait-disorder. Regional plots were created by using LocusZoom.

### Genetic instruments for sIL6R

We analyzed variants in *cis* up to 250 kb around *IL6R*. We selected IVs as previously preconized^39–41^ by selecting independent gene variants (*r*^2^ < 0.1 in the European 1000 Genome Project reference panel) significantly associated with sIL6R plasma level and with a *F*-statistic > 15 for MR analyses (Supplementary Table 1). We identified 34 IVs associated with sIL6R circulating levels. There was no IV located in exon of IL6R. For stroke phenotypes (AS, AIS, LAS, and SVS), RA and asthma, there were 33, 30, and 28 IVs available for analyses (Table 1, Supplementary Table 1). *F*-statistics for each instrument was estimated by *F* = beta^2^/SE^2^ ^42,43^ (Supplementary Table 1). Functional variant annotation was performed with ANNOVAR^44^ using default parameter settings (Supplementary Table 1).

### Mendelian randomization

A two-sample MR study was performed using the Mendelian Randomization package. Random-effects inverse variance-weighted (IVW) MR was used to determine causal effects of sIL6R on different traits and disorders. This approach assumes that the outcomes are affected only by the exposure and assign the intercept at zero.^45,46^ Different sensitivity measures were performed. Horizontal pleiotropy was assessed with Egger MR using the inclusion of the intercept in the regression analysis.^46^ A *P*_intercept_ < 0.05 was considered as significant for the presence of horizontal pleiotropy. Weighted median method was used to strengthen causal inference results.^19^ A *P*_causal_ < 0.05 was considered as significant. We also performed two-sample MR analyses without rs4129267, the IV with the lowest *p*-value for its association with sIL6R level. This analysis was performed to exclude the possibility that the association is driven by one gene variant with a large effect size. Multivariable MR was performed using the Mendelian Randomization package to determine if confounders (atrial fibrillation) mediated the causal association between sIL6R and the outcomes (cardio-embolic strokes and any strokes). An association was deemed causal when *P*_causal _ < 0.05. As previously described,^20^ we performed colocalization analyses by using Coloc.^47^ A region spanning ± 250 kb was included in the analyses and a PP4 > 80% was considered as a positive colocalization signal between the pQTL (sIL6R) and genetic association data for the disease. Colocalization analyses were conducted using the “coloc” R package. Statistical analyses were performed with R version 3.5.1. Two-sided *P* values below 0.05 were considered significant.

### Genetic correlation analyses

We estimated genetic correlation for RA with AF, stroke and CAD using cross-trait LD score regression with summary statistics.^48^ Gene variants in the major histocompatibility complex (MHC) (chr6: 26Mb-34Mb) were not included as well as those with extreme effect size (chi^2^ statistic > 80) and without match in the 1000 Genomes dataset. European LD score data from the 1000 Genomes were used. The analysis was performed with LD Hub.^49^

### Phenome-wide association study

The top variant for association with circulating sIL6R (rs4129267) was assessed in a PheWAS by using individual data from UKB (*n* = 353,378 unrelated individuals of European ancestry). We curated 832 phenotypes from anthropometric traits, health questionnaires, ICD10 diagnostic codes, OPCS-4 procedure codes, imaging data, and laboratory markers. Continuous variables were examined individually to exclude outlier values and quantile normalization was applied. For each phenotype, an additive logistic (binary phenotype) or linear regression (continuous phenotype) test was performed, adjusting for age, sex and the first 10 ancestry-based principal components using SNPTEST v2.5.2.^50^ Results for each phenotype were plotted using the PheWAS R package. The codes and official description of disorders and traits are provided in Supplementary Table 5. A significance threshold of *P* = 6.0 × 10^−5^ (0.05/832) was applied to correct for multiple testing (Bonferroni correction).

### S-PrediXcan analysis

Gene expression level and its association with disorders was assessed by using S-PrediXcan, which uses summary-level data statistics and expression quantitative trait loci (eQTL) in different tissues.^51,52^ S-PrediXcan was applied to GWAS summary data statistics for cardiovascular-immune disorders and using prediction models derived from GTExV7. Bonferroni correction was applied to identify significant *IL6R* tissue expression-disease pair associations (*P* *<* 0.001; 0.05/48 tissues).

### Reporting summary

Further information on research design is available in the Nature Research Reporting Summary linked to this article.

## Supplementary information

Supplementary Informations

Reporting Summary

## Data Availability

All GWAS summary statistics are available online: sIL6R and SAA (http://www.phpc.cam.ac.uk/ceu/proteins/), CRP (https://www.ebi.ac.uk/gwas/downloads/summary-statistics). AF (https://www.ebi.ac.uk/gwas/downloads/summary-statistics), Stroke (http://www.megastroke.org/download.html), CAD (https://www.ebi.ac.uk/gwas/downloads/summary-statistics), CARDIOGRAMplusC4D (http://www.cardiogramplusc4d.org/data-downloads/). AAA, HDL, LDL, total cholesterol and triglycerides (http://www.ukbiobank.ac.uk/). RA (https://grasp.nhlbi.nih.gov/FullResults.aspx), AD (https://grasp.nhlbi.nih.gov/FullResults.aspx). Asthma (https://www.ebi.ac.uk/gwas/downloads/summary-statistics). Fathers/mothers age at death (http://ldsc.broadinstitute.org/ldhub/).
