# Supplementary material for: A Mendelian randomization study of IL6 signaling in cardiovascular diseases, immune-related disorders and longevity
Source: NPJ Genom Med. 2019 Sep 20;4:23. doi: 10.1038/s41525-019-0097-4 (PMC6754413; doi:10.1038/s41525-019-0097-4)
Supplement: Supplementary file 1 — Supplementary Informations [file 41525_2019_97_MOESM1_ESM.pdf]

Supplementary figure 1

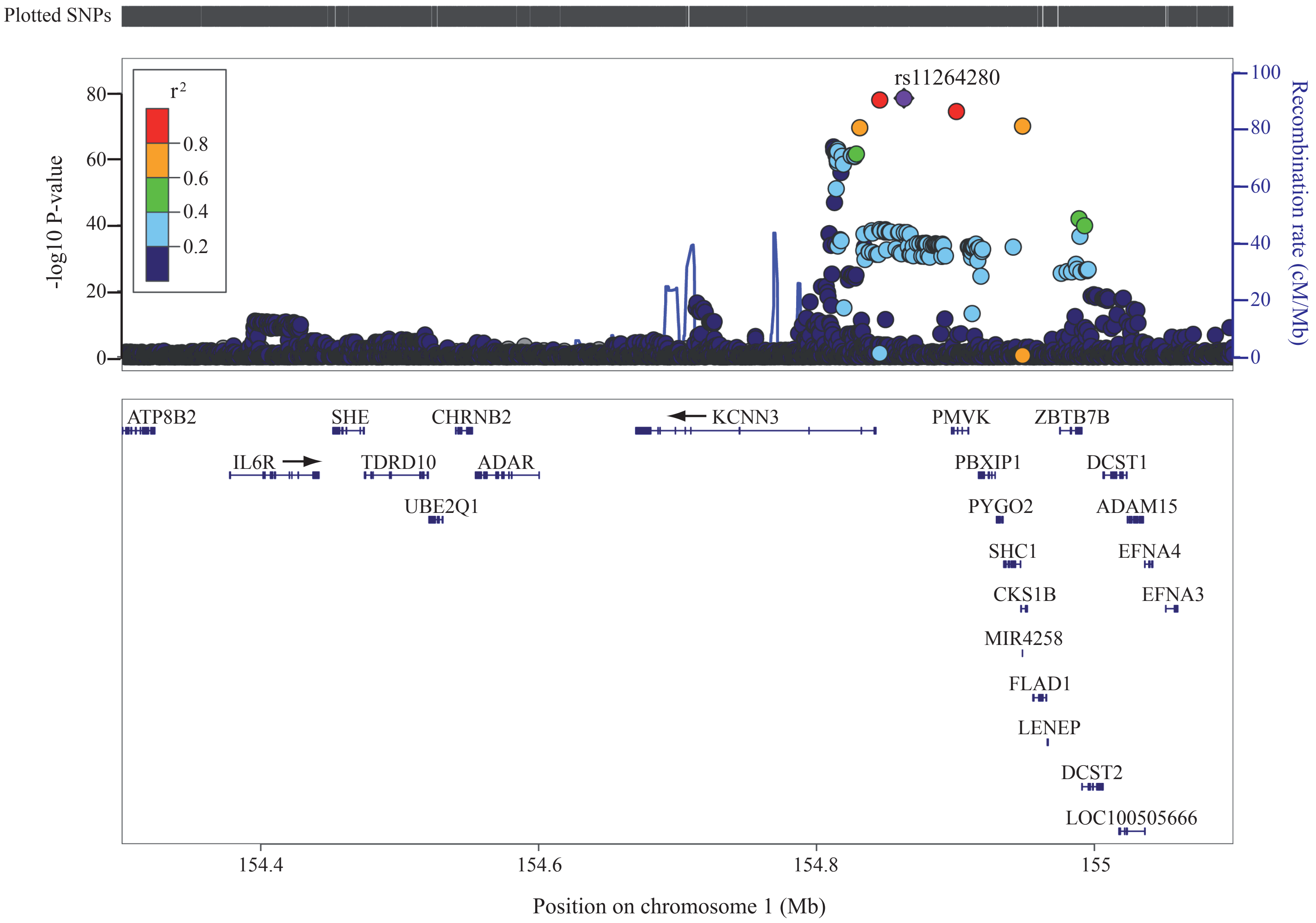

Supplementary Figure 1: Regional plot centered on KCNN3. Regional plot of variants associated with AF. Genes located 800 kb up- and downstream of *KCNN3* are displayed below the plot.

Supplementary Table 1 : Genetic variants used as instrumental variables for Mendelian Randomization analyses

Chr	Position	SNP_ID	EA	NEA	Beta	SE	p-value	*F* -statistic	Functionnal annotation	Mapped gene
1	154426264	rs4129267	T	C	1.1148	0.0157	7.41 x 10-1101	5041.90	intronic	IL6R
1	154507899	rs4633282	T	C	0.6124	0.0286	6.31 x 10-102	458.50	intronic	TDRD10
1	154505106	rs77994623	T	C	-0.6184	0.0307	2.75 x 10-90	405.75	intronic	TDRD10
1	154391882	rs35717427	A	G	0.5238	0.036	5.62 x 10-48	211.70	intronic	IL6R
1	154568086	rs11264224	A	C	-0.4576	0.0336	3.89 x 10-42	185.48	intronic	ADAR
1	154394297	rs7525477	A	G	-0.3502	0.0261	4.79 x 10-41	180.03	intronic	IL6R
1	154571826	rs3766924	T	C	-0.3863	0.0300	7.94 x 10-38	165.81	intronic	ADAR
1	154342517	rs79438587	T	C	0.405	0.0338	4.07 x 10-33	143.57	intergenic	ATP8B2
1	154461480	rs41269913	T	C	0.6983	0.0599	1.95 x 10-31	135.90	intronic	SHE
1	154538917	rs67860750	C	G	0.3985	0.0353	1.41 x 10-29	127.44	intergenic	CHRNB2
1	154415675	rs79219014	T	G	0.7582	0.0767	4.57 x 10-23	97.72	intronic	IL6R
1	154516404	rs76289529	T	C	0.6656	0.0678	9.77 x 10-23	96.38	intronic	TDRD10
1	154627213	rs10752605	A	G	-0.3349	0.0374	3.63 x 10-19	80.18	intergenic	ADAR
1	154409434	rs79778789	A	G	-0.7852	0.0887	8.32 x 10-19	78.36	intronic	IL6R
1	154154587	rs61806853	T	C	-0.4957	0.0573	5.01 x 10-18	74.84	intronic	TPM3
1	154420333	rs113580743	A	G	-0.5141	0.0605	1.95 x 10-17	72.21	intronic	IL6R
1	154510155	rs116805289	A	C	0.6267	0.0822	2.51 x 10-14	58.13	intronic	TDRD10
1	154534480	rs149551556	T	C	-0.6651	0.0947	2.19 x 10-12	49.33	intergenic	UBE2Q1
1	154443014	rs79925547	T	C	0.752	0.1155	7.41 x 10-11	42.39	intergenic	IL6R
1	154564712	rs3766925	A	T	0.184	0.0294	3.89 x 10-10	39.17	intronic	ADAR
1	154270877	rs3103309	T	C	0.16	0.0261	8.51 x 10-10	37.58	intergenic	HAX1
1	154454309	rs76518735	A	C	0.5646	0.0924	1.00 x 10-9	37.34	UTR3	SHE
1	154416805	rs139952834	T	C	-0.6506	0.1072	1.29 x 10-9	36.83	intronic	IL6R
1	154320942	rs2297607	A	G	0.1756	0.0291	1.66 x 10-9	36.41	exonic	ATP8B2
1	154432342	rs77741705	C	G	0.5205	0.0941	3.16 x 10-8	30.60	intronic	IL6R
1	154431123	rs142712385	A	T	-0.2782	0.0534	1.91 x 10-7	27.14	intronic	IL6R
1	154541717	rs138398618	A	G	-0.5044	0.1027	9.12 x 10-7	24.12	intronic	CHRNB2
1	154624636	rs147745605	T	C	0.5012	0.1034	1.26 x 10-6	23.50	intergenic	ADAR
1	154517689	rs115697580	A	G	-0.4684	0.0967	1.26 x 10-6	23.46	intronic	TDRD10
1	154585644	rs115880387	A	G	-0.6209	0.1388	7.59 x 10-6	20.01	intronic	ADAR
1	154339794	rs116568035	A	G	-0.3112	0.0696	7.76 x 10-6	19.99	intergenic	ATP8B2
1	154445362	rs147700711	T	G	-0.4972	0.119	2.95 x 10-5	17.46	intergenic	IL6R
1	154334023	rs56258967	T	C	0.4718	0.1151	4.17 x 10-5	16.80	intergenic	ATP8B2
1	154266792	rs181862028	A	T	-0.4133	0.1042	7.24 x 10-5	15.73	intergenic	HAX1

Supplementary Table 2 : Association between sIL6R and CAD (CARDIOGRAMplusC4D) in multivariate MR corrected for lipids

	OR	95% Confidence Interval		Pcausal
**HDL**	0.947	0.933	0.961	< 0.0001
**Total Cholesterol**	0.947	0.932	0.962	< 0.0001

Supplementary Table 3 : Sensitivity analyses for cardiovascular and immune disorders, blood parameters and longevity

					MR-Egger				
**Phenotypes**	**Cases**	**Controls**	**Ivs**	**SNPs not available for analyses**	**OR**	**95% Confidence Interval**		**Pegger**	**Pintercept**
**AF**	60,620	970,216	34	-	0.952	0.933	0.970	4.82 x 10-7	0.224
**Any Stroke**	40,585	406,111	33	rs56258967	0.979	0.954	1.005	0.109	0.889
**LA-Stroke**	4,373	406,111	33	rs56258967	0.993	0.933	1.058	0.832	0.345
**SV-Stroke**	5,386	406,111	33	rs56258967	0.972	0.916	1.031	0.340	0.395
**AI-Stroke**	34,217	406,111	33	rs56258967	0.977	0.951	1.005	0.107	0.943
**CE-Stroke**	7,193	406,111	34	-	0.932	0.873	0.994	0.032	0.527
**CAD**	122,733	424,528	34	-	0.959	0.947	0.971	9.23 x 10-11	0.315
**AAA**	821	352,557	34	-	0.821	0.731	0.922	8.86 x 10-4	0.577
**RA**	18,136	49,724	30	rs79219014, rs147745605, rs56258967, rs181862028	0.939	0.889	0.992	0.025	0.504
**AD**	18,900	84,166	34	-	1.080	1.037	1.125	2.29 x 10-4	0.341
**Asthma**	180,129	180,709	28	rs79778789, rs77741705, rs115880387, rs147700711, rs56258967, rs181862028	1.036	1.014	1.058	1.22 x 10-3	0.539
**Blood Parameters**	**Participants**		**Ivs**	**SNPs not available for analyses**	**Beta**	**95% Confidence Interval**		**Pegger**	**Pintercept**
**HDL**	255,929		34	-	0.005	-0.004	0.014	0.300	0.911
**LDL**	279,367		34	-	0.002	-0.005	0.008	0.622	0.877
**Total cholesterol**	279,913		34	-	0.005	-0.001	0.012	0.117	0.824
**Triglycerides**	279,686		34	-	0.004	-0.002	0.010	0.192	0.027
**CRP**	9,961		34	-	-0.099	-0.142	-0.056	5.64 x 10-6	0.887
**SAA**	3,301		34	-	0.015	-0.049	0.079	0.641	0.171
**Longevity**	**Participants**		**Ivs**	**SNPs not available for analyses**	**Beta**	**95% Confidence Interval**		**Pegger**	**Pintercept**
**Fathers age at death**	248,726		34	-	0.007	0.001	0.014	0.032	0.872
**Mothers age at death**	199,690		34	-	0.006	-0.002	0.014	0.116	0.561

Supplementary Table 3 (continued) : Sensitivity analyses for cardiovascular and immune disorders, blood parameters and longevity

	Weighted Median				Whitout rs4129267			
**Phenotypes**	**OR**	**95% Confidence Interval**		**Pcausal**	**OR**	**95% Confidence Interval**		**Pcausal**
**AF**	0.964	0.953	0.975	1.05 x 10-10	0.959	0.947	0.972	4.37 x 10-10
**Any Stroke**	0.979	0.964	0.995	8.91 x 10-3	0.976	0.959	0.993	6.83 x 10-3
**LA-Stroke**	0.977	0.938	1.018	0.269	0.955	0.916	0.997	0.034
**SV-Stroke**	0.956	0.920	0.993	0.019	0.944	0.907	0.983	5.14 x 10-3
**AI-Stroke**	0.980	0.963	0.997	0.018	0.974	0.956	0.993	5.88 x 10-3
**CE-Stroke**	0.956	0.925	0.988	7.10 x 10-3	0.942	0.901	0.985	7.98 x 10-3
**CAD**	0.965	0.957	0.973	1.54 x 10-16	0.963	0.955	0.972	2.49 x 10-16
**AAA**	0.848	0.781	0.920	7.29 x 10-5	0.842	0.783	0.907	5.30 x 10-6
**RA**	0.941	0.917	0.966	5.03 x 10-6	0.977	0.938	1.018	0.268
**AD**	1.069	1.040	1.099	2.01 x 10-6	1.055	1.024	1.086	3.49 x 10-4
**Asthma**	1.032	1.023	1.042	3.65 x 10-11	1.027	1.012	1.041	2.93 x 10-4
**Blood Parameters**	**Beta**	**95% Confidence Interval**		**Pcausal**	**Beta**	**95% Confidence Interval**		**Pcausal**
**HDL**	0.005	0.001	0.010	0.019	0.005	-0.001	0.011	0.083
**LDL**	0.003	-0.001	0.008	0.131	0.001	-0.003	0.005	0.673
**Total cholesterol**	0.007	0.002	0.011	0.004	0.003	-0.001	0.007	0.125
**Triglycerides**	0.001	-0.004	0.005	0.792	-0.004	-0.008	0.000	0.069
**CRP**	-0.091	-0.120	-0.062	7.07 x 10-10	-0.107	-0.131	-0.083	1.62 x 10-18
**SAA**	-0.009	-0.050	0.031	0.648	-0.044	-0.084	-0.003	0.034
**Longevity**	**Beta**	**95% Confidence Interval**		**Pcausal**	**Beta**	**95% Confidence Interval**		**Pcausal**
**Fathers age at death**	0.006	0.001	0.011	0.010	0.008	0.003	0.012	4.69 x 10-4
**Mothers age at death**	0.005	-0.001	0.010	0.086	0.011	0.006	0.016	5.19 x 10-6

Supplementary Table 4 : Colocalization analyses (500 kpb window arround rs4129267)

	PP4 (%)
**AF**	31.5
**Any Stroke**	10.9
**LA-Stroke**	3.29
**SV-Stroke**	16.8
**AI-Stroke**	13.9
**CES-Stroke**	2.71
**CAD**	6.01
**AAA**	88
**RA**	53.9
**AD**	98
**Asthma**	99.2
**Longevity (fathers age at death)**	17.6
**Longevity (mothers age at death)**	3.58

Supplementary Table 5 : Description and codes of the significant phenotypes of the PheWAS for rs4129267

Phenotype	Description	Code(s)
CABG or PCI	CABG or PCI procedure (OPCS-4)	K40-K51, K75
coronary artery bypass	CABG procedure (OPCS-4)	K40-K51
angina	Angina pectoris (ICD10)	I20
ischemic heart diseases	Ischemic heart diseases (ICD10)	I20-I25
eczema dermatitis	Eczema/dermatitis, self-reported	Non-cancer illness 1452
dermatology	Dermatology diagnoses, self-reported	Non-cancer illness 1344, 1345, 1452-1455, 1548-1550, 1625, 1660, 1661, 1667, 1680
dermatitis and eczema	Dermatitis and eczema (ICD10)	L20-L30
monocyte count	Result of "Monocytes Number" assay, performed on blood sample, obtained from UK Biobank assessment centre visit. Monocyte count is the proportion of (monoctyes / 100) x white blood cell count.	Data-Field 30130
mean platelet volume	Result of "Mean Platelet Volume" assay, performed on blood sample, obtained from UK Biobank assessment centre visit. Mean Platelet Volume is the average volume of individual platelets derived from the platelet histogram.	Data-Field 30100
mean corpuscular volume	Result of "Mean Corpuscular Volume" assay, performed on blood sample, obtained from UK Biobank assessment centre visit. Mean Corpuscular Haemoglobin Concentration (pg) is the weight of hemoglobin in the average erythrocyte, computed by the formula: MCH = (hemoglobin/red blood cells) x 10.	Data-Field 30040
red blood cell distribution	Result of "Red Distribution Width" assay, performed on blood sample, obtained from UK Biobank assessment centre visit. Red blood cell Distribution Width is the size distribution spread of the erythrocyte population derived from the red blood cell histogram. It is the coefficient of variation (CV) expressed in % of the red blood cell size distribution.	Data-Field 30070
platelet count	Result of "Platelet Count" assay, performed on blood sample, obtained from UK Biobank assessment centre visit. Platelet count is the number of thrombocytes derived from the platelet histogram.	Data-Field 30080
haemoglobin concentration	Result of "Haemoglobin Concentration" assay, performed on blood sample, obtained from UK Biobank assessment centre visit.	Data-Field 30020
hayfever allergic	ACE touchscreen question "Has a doctor ever told you that you have had any of the following conditions?" Hayfever, allergic rhinitis or eczema	Data-Field 6152
asthma	ACE touchscreen question "Has a doctor ever told you that you have had any of the following conditions?" Asthma	Data-Field 6152
